# Development of Microsatellite Markers and Analysis of Genetic Diversity and Population Structure of *Colletotrichum gloeosporioides* from Ethiopia

**DOI:** 10.1371/journal.pone.0151257

**Published:** 2016-03-15

**Authors:** Asmare D. Moges, Belayneh Admassu, Derbew Belew, Mohammed Yesuf, Joyce Njuguna, Martina Kyalo, Sita R. Ghimire

**Affiliations:** 1 Department of Horticulture, Ethiopian Institute of Agricultural Research, Adama, Ethiopia; 2 Department of Agricultural Biotechnology, Ethiopian Institute of Agricultural Research, Holetta, Ethiopia; 3 Department of Horticulture and Plant Sciences, College of Agriculture and Veterinary Medicine, Jimma University, Jimma, Ethiopia; 4 Biosciences eastern and central Africa Hub, International Livestock Research Institute, Nairobi, Kenya; United States Department of Agriculture, UNITED STATES

## Abstract

Twenty three polymorphic microsatellite markers were developed for citrus plant pathogenic fungus, *Colletotrichum gloeosporioides*, and were used to analyze genetic diversity and population structure of 163 isolates from four different geographical regions of Ethiopia. These loci produced a total of 118 alleles with an average of 5.13 alleles per microsatellite marker. The polymorphic information content values ranged from 0.104 to 0.597 with an average of 0.371. The average observed heterozygosity across all loci varied from 0.046 to 0.058. The gene diversity among the loci ranged from 0.106 to 0.664. Unweighted Neighbor-joining and population structure analysis grouped these 163 isolates into three major groups. The clusters were not according to the geographic origin of the isolates. Analysis of molecular variance showed 85% of the total variation within populations and only 5% among populations. There was low genetic differentiation in the total populations (F_ST_ = 0.049) as evidenced by high level of gene flow estimate (N_m_ = 4.8 per generation) among populations. The results show that Ethiopian *C*. *gloeosporioides* populations are generally characterized by a low level of genetic diversity. The newly developed microsatellite markers were useful in analyzing the genetic diversity and population structure of the *C*. *gloeosporioides* populations. Information obtained from this study could be useful as a base to design strategies for better management of leaf and fruit spot disease of citrus in Ethiopia.

## Introduction

Citrus (*Citrus* spp.) are economically important fruit crops in Ethiopia. The sustainable production of citrus in Ethiopia is severely constrained by various fungal diseases including *C*. *gloeosporioides*, *Phythophtora* spp., *Alternaria* spp., *Penicillium* spp. and *Pseudocercospora angolensis* [1–3]. In Ethiopia, many farmers have reported complete losses of fruit production and given-up the citrus cultivation [3,4]. Therefore, identification of sources of resistance and epidemiological studies has been recognized as the priority areas of citrus research in Ethiopia [3]. Diseases resistant varieties could serve as essential part of sustainable and long-term disease management strategies [5]. However, resistance breeding requires extensive information on the genetics of host-pathogen interactions [6].

Members of genus *Colletotrichum* represent a group of plant pathogenic fungi that can infect a wide range of plant species including many commercially cultivated crops [7,8]. *Colletotrichum gloeosporioides* sensu lato is one of the most common and widely distributed plant pathogens worldwide [9,10]. It is an important pathogen associated with more than 470 different host species, either as a primary disease causing organism, or isolated from deteriorated plant parts [11]. It is economically important pathogen of a wide range of fruit crops, including citrus [12–15], apple [16], avocado [17], mango [18], olive [19], papaya [20], passion fruit [18] and strawberry [21–23]. *Colletotrichum gloeosporioides* has been found associated with the leaf and fruit disease of citrus across citrus growing regions of Ethiopia. Therefore, it is vital to understand the genetic diversity and population structure of the fungus, and define the regional populations of the pathogen to develop and implement effective disease management strategies [24].

DNA markers are in wide use for analyzing the dynamics of plant pathogen populations due to their high precision levels [25]. They are rapid, highly specific and can be detected using minute quantity of DNA [26,27]. Microsatellites [28], also known as simple sequence repeats (SSR) [29] or short tandem repeats (STR) [30], are one of the highly versatile genetic markers that have been widely used for the genetic study of plant pathogens [27,31]. Microsatellites comprise tandemly repeated nucleotide motifs of one to six base pairs long [31–35]. They are relatively abundant, co-dominant, ubiquitous, and exhibit extensive levels of polymorphisms in prokaryotic and eukaryotic genomes [29,36–41]. Microsatellites are found in both coding and non-coding regions [34,39,40,42,43], but they are more abundant in non-coding regions [31,35,44]. Microsatellites enable higher statistical power and discrimination among genotypes [45]. The high levels of polymorphisms observed in SSR markers and the relative ease of detection of these polymorphisms by PCR amplification has led to the wide applications of SSRs as genetic markers [46]. Microsatellites have proven to be invaluable in many fields of biology that span from forensic DNA studies to genome mapping, paternity testing, population genetics and biological resources conservation [33,47–49]. One of the major limitations of SSR markers is high mutation rates ranging from 10^−3^ to 10^−6^ per generation [50–52] due to slipped-strand miss-pairing and subsequent resulting errors during DNA replication, repair and recombination [53–55]. The major drawbacks of the traditional methods to develop SSR markers are that they require prior knowledge of the DNA sequences of the flanking regions, they are expensive and time-consuming, and they have low throughput due to difficulties for automation and data management [35,41,56,57]. A high number of microsatellites loci are also required for a reliable phylogenetic reconstruction [31]. At present, these problems are partly resolved with the advent of high-throughput next-generation sequencing technologies and multiplexing microsatellites [31,58].

Fungal genomes appear to contain fewer SSR sequences than other eukaryotes [59]. However, when polymorphic loci are available, they can be very useful for genome mapping, and genetic diversity and population genetic studies [60]. Accordingly, SSRs have been used for the study of the genetic diversity of various plant pathogenic fungi including *Ascochyta rabiei* [61], *Ceratocystis fimbriata* [62], *Macrophomina phaseolina* [63], *Puccinia graminis* and *P*. *triticina* [6,64,65], *Sclerotinia subarctica* and *S*. *sclerotiorum* [66], and *C*. *gloeosporioides* [24]. Despite the importance of *C*. *gloeosporioides* on citrus, its population genetic diversity has not been studied. Therefore, the objective of this study was to develop microsatellite markers and analyze the genetic diversity and population structure of *C*. *gloeosporioides* populations from the major citrus production areas of Ethiopia.

## Materials and Methods

Only infected citrus leaf and fruit samples were collected from both private and public owned citrus orchards/farms. We confirm that the owners of each private orchard and the farm manager of each commercial public citrus farm gave permission to collect the samples in each site. We also confirm that the field sample collection did not involve any endangered or protected species.

### Fungal isolates and culture conditions

A total of 163 *C*. *gloeosporioides* isolates were recovered on water agar from symptomatic citrus leaf and fruit samples collected from major citrus production areas of Ethiopia during 2012 to 2014. Single spore or hyphal tip cultures developed in water agar and subsequently transferred to potato dextrose agar (PDA; Oxoid, UK) supplemented with antibiotics (100 ppm of ampicillin, 50 ppm of chloramphenicol and 50 ppm of streptomycin sulphate). Isolates were assigned into four populations based on their geographical origin (Table 1). The details of isolates used in this study are presented in supplementary S1 Table.

**Table 1:** Geographic origins of *Colletotrichum gloeosporioides* populations, the number of isolates represented in each population and their hosts.

Region	No. of isolates	Origin (Districts)	Host	Year of collection
Central	43			2012–2014
		Abeshge	*Citrus sinensis*	
		Cheha	*C*. *sinensis*	
		Geta	*C*. *sinensis*	
		Gorro	*C*. *sinensis*	
		Kebena	*C*. *sinensis*	
		Sekoru	*C*. *sinensis* and *C*. *reticulata*	
		Wolliso	*C*. *sinensis* and *C*. *aurantium*	
Northwest Ethiopia	43			2013
		Guangua	*C*. *sinensis*	
		Jabitehnan	*C*. *sinensis* and *C*. *reticulata*	
South Ethiopia	30			2013–2014
		Abaya	*C*. *sinensis* and *C*. *aurantium*	
		Aleta Wendo	*C*. *sinensis*	
		Boloso Sore	*C*. *sinensis*	
		Damot Pulasa	*C*. *sinensis*	
Southwest Ethiopia	47			2013
		Debre Werk	*C*. *sinensis*	
		Ginbo	*C*. *sinensis*	
		Gomma	*C*. *sinensis*	
		Jimma	*C*. *sinensis*	
		Mana	*C*. *sinensis*	
		Shebe Senbo	*C*. *sinensis*	
Total	163			

### DNA extraction

The total genomic DNA was extracted from lyophilized mycelia obtained from 10-day-old cultures grown on PDA as described by Ghimire *et al*. [67] using Qiagen MagAttract96 DNA Plant Core Kit according to the manufacturer’s instructions. The quality and concentration of extracted DNA were estimated using the NanoDrop 2000c Spectrophotometer (Thermo Fisher Scientific, Walthum, MA) and visualized in 1% agarose gel stained with SYBR Safe DNA gel stain under ultra violet-light (UVP BioImaging Systems, Upland, CA). DNA was stored at -20°C until further use.

### Genome sequencing and assembly

Whole genome DNA libraries were constructed using next-generation Illumina MiSeq sequencing technology according to the manufacturer’s instructions for five representative *C*. *gloeosporioides* isolates (016, 064, 092, 193 and 194). The libraries were sequenced and 301 bp x 2 paired-end reads were obtained. Quality and nucleotide distribution of the sequences were explored using FASTX Toolkit (http://hannonlab.cshl.edu/fastx_toolkit/) and FastQC version 0.11.2 (http://www.bioinformatics.babraham.ac.uk/projects/fastqc/) installed on a high performance computing (hpc) Linux server. The adapters indexed at the beginning and end of each sequence were removed using Scythe version 0.994-beta (https://github.com/vsbuffalo/scythe). Poor quality sequences at both ends were trimmed and the reads with ‘N’s were filtered out using SolexaQA++ version 3.1.2 (http://solexaqa.sourceforge.net) [68]. The genome was assembled with SPAdes version 3.1.1 [69] genome assembler program using the trimmed and filtered reads. The assembled sequences were used for SSR markers development.

### SSR identification and primer design

Genome-wide SSR markers database was developed for *C*. *gloeosporioides*. Instead of trying to design primers directly from the Illumina reads, genome assembly was performed to achieve longer contiguous sequences followed by microsatellite search and primer design. SSR motifs were identified using MIcroSAtellite identification tool (MISA) [70], simple sequence repeat locator (SSRLocator) version 1 [71], and a web-based simple sequence repeat identification tool (SSRIT) [72]. The results obtained using these three search tools were compared and validated using tandem repeats finder (TRF) version 4.07b (http://tandem.bu.edu/trf/trf.advanced.submit.html) [73]. The outputs were processed using a custom PERL script modified from “P3in.pl” (http://pgrc.ipkgatersleben.de/misa/primer3.html) and primers were designed for selected SSR loci using Primer3 [74] in a batch mode on hpc Linux server and validated using Primer Premier version 6.22 (PREMIER Biosoft package, Palo Alto, CA) software. The parameters for primer design were: product size of 70 to 300 bp; primer size of 18 to 22 bp with optimal length 20 bp; primer melting temperature (Tm) of 50°C to 60°C with an optimum at 55°C; and primers were at least 5 bp away from the SSR locus. PrimerDigital (http://primerdigital.com/tools/) [75], a Java based tool with multiple options was used to predict the annealing temperature and the amplified products for each primer pair.

### PCR amplification and genotyping

Microsatellite markers were developed and tested on 13 geographically representative *C*. *gloeosporioides* isolates (032, 050, 092, 111, 114, 116, 129, 153, 189, 193, 194, 197 and 198) for polymorphism level. The 5' end of the forward primers of all SSR loci were labeled with fluorescent dyes (6-FAM = blue, PET = red, VIC = green, and NED = yellow).

The PCR amplification for each SSR loci was performed in standard PCR to determine the appropriate annealing temperature. PCR amplifications were performed in a total volume of 10 μl containing AccuPower PCR PreMix without dye (Bioneer, Daejeon, Republic of Korea), additional 0.5 mM MgCl_2_ (Promega, Venlo, The Netherlands), 0.05 to 0.15 μM forward primer, 0.05 to 0.15 μM reverse primer, and 2.0μl template DNA (20 ng/μl). PCR reaction without template DNA was used as control. Amplifications were performed in a GeneAmp PCR System 9700 thermocycler (Applied Biosystems, Foster City, CA) using the following PCR cycling conditions: initial denaturation step at 94°C for 3 min; followed by 30 cycles of denaturation at 94°C for 30 sec, annealing at 50°C, 51°C or 54°C for 1 min, and extension at 72°C for 2 min; and a final extension step at 72°C for 10 min. To reveal polymorphism and allele identification, the PCR products were separated in 2% agarose gel stained with GelRed and visualized under UV light. The sizes of the PCR amplicons were estimated using a 100-bp ladder (Invitrogen, Carlsbad, CA).

In addition to agarose gel electrophoresis, SSR fragment sizes and allele variations in the repeats were assessed by capillary electrophoresis of amplicons and by sequencing the amplified loci. The multiplexed PCR products were mixed with 8.87 μl Hi-Di formamide and 0.135 μl fluorescent-labeled GeneScan™-500 LIZ size standard (Applied Biosystems, Warrington, UK) in a 96-well microtiter plate. The mixed products were denatured at 95°C for 3 min and snap-chilled on ice for 5 min. The products were then electrophoresed using an ABI PRISM 3730xl automated sequencer (Applied Biosystems, Foster City, CA). Allele size scoring of the fragments was manually performed using GeneMapper software version 4.1 (Applied Biosystems, Foster City, CA). The SSR markers were scored for the presence or absence of the corresponding bands among the test isolates.

### SSR polymorphism and genetic diversity

Polymorphic SSR markers were used to analyze the genetic diversity of 163 Ethiopian *C*. *gloeosporioides* isolates. The basic statistics, such as the major allele frequency, number of alleles per locus, gene diversity, heterozygosity, and polymorphic information content (PIC) were determined using PowerMarker version 3.25 [76]. For each SSR marker, the degree of polymorphism estimated by gene diversity [77] was calculated for all the 163 isolates. To estimate the discriminatory power of the microsatellite loci, the PIC for each locus was computed by *PIC* = 1 − ∑*Pi*^2^, where *Pi*^2^ referred to the sum of the *i*th allelic frequency of each microsatellite locus for the genotypes [78,79].

The number of different alleles per locus, number of effective alleles per locus, number of private alleles, observed heterozygosity, expected heterozygosity [80], and Shannon's Information Index were computed for each population using GenAlEx version 6.501 [81]. Allelic richness and private allelic richness were computed using the rarefaction method [82] implemented in HP-Rare version 1.1 software [83]. The exact tests to estimate the deviation from Hardy-Weinberg equilibrium (HWE) and genotypic linkage disequilibrium for all pairs of loci were computed using GenePop version 4.3 [84] and corrected using the sequential Bonferroni procedure [85].

### Population structure and gene flow

The Sokal and Michener dissimilarity index was used to generate dissimilarity matrices [86], based on the set of SSR markers. To assess the distribution of gene diversity and estimate the components of variances of the populations, analysis of molecular variance (AMOVA) based on co-dominant SSR loci was computed using GenAlEx [81]. To investigate population differentiation, [87] fixation index (F_ST_) of the total populations and pairwise F_ST_ among all pairs of populations were computed, and significance was tested based on 1000 bootstraps. Principal Coordinate Analysis (PCoA) was done using the same software to show the pattern of genetic differentiation of the populations of *C*. *gloeosporioides* isolates. Gene flow among populations was estimated using indirect method based on the number of migrants per generation (N_m_) using the formula, N*m* = 0.25(1 − F*st*)/F*st* [88].

Frequency-based genetic distances were calculated using shared alleles distance matrix [89], and used to construct Unweighted Neighbor-joining dendrogram for the 163 isolates belonging to the four populations of *C*. *gloeosporioides* using DARwin version 6.0.010 (http://darwin.cirad.fr) [90]. The resulting tree was bootstrapped with 1000 replicates [91] and viewed using TreeView version 1.6.6 [92; available at http://taxonomy.zoology.gla.ac.uk/rod/rod.html].

The pattern of population structure and detection of admixture were inferred using a Bayesian model-based clustering algorithm implemented in STRUCTURE version 2.3.4 [93,94] using the SSR loci data. For this, two separate analyses were run with and without prior information about the populations. The first was done by assigning the site of collection as the putative population origin for each individual and the second run was without giving such information and letting the STRUCTURE software assign each individual into a population. The admixture model with correlated allele frequencies was used as suggested in the manual. To determine most appropriate number of populations (*K*), a burn-in period of 25,000 was used in each run, and data were collected over 100,000 Markov Chain Monte Carlo (MCMC) replications from *K* = 1 to *K* = 10. The probability values were averaged across runs for each cluster. This procedure clusters individuals into populations and estimates the proportion of membership in each population for each individual [93]. The *K* value was determined by the log probability of data (Ln P(D)) based on the rate of change in LnP(D) between successive *K*. The optimum *K* value was predicted following the simulation method of Evanno *et al*. [95] using the web-based software STRUCTURE HARVESTER version 0.6.92 [96].

## Results

### Marker development

A total of 50 SSR markers (S2 Table) were used for evaluating allelic diversity in *C*. *gloeosporioides* isolates. Multiplex PCR amplifications were performed using four to five SSR primer pairs in each PCR based on annealing temperatures (S3 Table). Forty-six SSR primer pairs produced clear single amplicons while four primer pairs (CG12, CG24, CG38 and CG41) did not amplify. Due to resource limitations, twenty-eight SSR loci with 50°C and 51°C annealing temperatures were tested on the 13 *C*. *gloeosporioides* isolates representing different geographic origins. Twenty-three (Table 2) of the SSR markers were polymorphic and demonstrated allelic diversity among the 13 test isolates. These 23 polymorphic SSR markers were used in genetic diversity analysis of 163 Ethiopian *C*. *gloeosporioides* isolates originated from four geographical regions.

**Table 2:** Characteristics of 23 polymorphic microsatellite markers developed in this study for population genetic diversity analysis of *Colletotrichum gloeosporioides* isolates.

Locus	Forward primer sequences (5’- 3’)	Reverse primer sequences (5’- 3’)	Ta (°C)*	Allele size range (bp)	Repeat motifs
CG1	CAAGCAGTCTTTCTGGTCTT	AAAACAACTTCTCTCGTCCA	51	125–129	(TG)6
CG2	TCACCTTCACTCACACTTGA	CTACTTCGAGACAAGCACG	51	201–203	(CT)6
CG3	GGTTTTCTCATTCTCAACA	CGACATGATCCATAGCAAG	50	247–253	(AT)6
CG4	AACTCAAGATCAAGAGCAGC	ATGTACAGACGCTCACACAA	51	150–161	(TG)6
CG6	AGAGCAAGACAGGTGGAATA	ATCCCTGACTGCATAAACC	51	201–220	(AC)6
CG7	ATCTCCAGAGAGAACACAGC	GAGACCTCACGGAATTGAC	51	158–187	(TG)7
CG9	GTCTTGATGCTGAAGTCCAC	CACTCCTTCATAGAACACCC	51	218–257	(TG)6
CG11	CAGTGAAGATAGGGAAGCAG	ACCACTCAGCGTATGAGAAA	51	107–152	(GT)8
CG14	ACATGACATCAAACCAGCTT	CTCTTGACCCGATGTTCTAT	51	173–179	(TC)7
CG16	CCATTCTTTGTACTGGTCGT	GACATCAGACATCCATCCTC	51	186–196	(TG)6
CG18	TCCAGACGGATAGCTTACAC	GAGGTATTGCGTCCACTAAG	51	197–204	(CT)7
CG20	CATAGTCCGTCCAGTCTCAT	CTAATGAAAAGTCGTGGAGC	51	218–235	(GA)8
CG21	GTCTCACTCAGTCTCAAGCC	AACACAGTCTGAGAGGCAAT	51	223–229	(AT)9
CG22	CTTCGAGTCACCTCTTCAAC	CAGAGTGGTAAAGGTGGTGT	51	158–241	(AC)7
CG23	TATTAGATCCCGACCTTGTG	ATCCTGGTCACCATAATCC	51	160–182	(GA)6
CG27	CCTGTTGATCCATGATGTAA	GAAAGGCTGACTTGTGAACT	50	126–138	(GT)6
CG28	CATATCTCTTCGTACCTCGC	GGTTTGTTGTCTGCTTCTCT	51	163–170	(AG)8
CG29	TTTCAACTACATCCCACCTC	GTATTTGAGGCTGAAGCAAG	51	55–74	(AC)7
CG30	CGTCATTTTCTGGATTCACT	ATCCATTGGGCTGTCCAT	50	137–163	(GT)9
CG32	TTGTTAGCATCGTGAGTCAG	GCAGTTGATTGAGCAGTACA	51	209–217	(AG)10
CG33	GGCATCTATGGACTAGCAGA	TCATACACCAAAGCTTCCTC	51	150–229	(GC)6
CG36	CCACTCAATTCAATGACAGA	TGAGAGAGTTGTGTCCATCA	50	225–265	(AC)7
CG37	TTATATGCCCCATACTCACC	GGGTCATCTTACACCGTTAC	50	214–231	(CA)8

* Ta = Annealing temperature

### SSRs polymorphism and gene diversity

The polymorphism and diversity of the different SSR loci are presented in Table 3. Availability of alleles in each locus (the proportion of loci without missing alleles) was one for all the loci. The 23 polymorphic SSR markers detected a total of 118 alleles in the 163 *C*. *gloeosporioides* isolates studied. Of the 118 alleles detected, 59 (50%) were rare (with frequency ≤ 0.05). The number of alleles per locus ranged from three to eight, with an average of 5.13 alleles. The allele size ranged from 55 to 265 bp (Table 2). The PIC values varied from 0.104 (CG29) to 0.597 (CG30), with an average of 0.371 per marker. Nine SSR loci were highly informative (PIC ≥ 0.5), seven were reasonably informative (0.5 <PIC > 0.25), and seven were slightly informative (PIC < 0.25). The frequency of the major allele in each locus varied from 0.41 to 0.94, with a mean of 0.69. The total multilocus microsatellite genotypes (N_G_) were 135, with a range of 3 to 9 for each microsatellite marker. The number of effective alleles was in the range of 1.0 to 2.3. The value of allelic richness (Rs) ranged from 1.25 to 3.01, with a mean of 2.26. Allelic richness is the number of alleles per locus which is a measure of genetic diversity indicative of a population's long-term potential for adaptability and persistence [97,98,99]. The private allelic richness (Rp) of the markers was in the range of 0.03 to 1.07. Private allelic richness is the number of unique alleles in a population. It is a simple measure of genetic distinctiveness [97]. Gene diversity, defined as the probability that two randomly chosen alleles from the population are different [100], varied from 0.106 (CG29) to 0.664 (CG30), with an average of 0.41. Low level of heterozygosity (0.000 to 0.043) was detected in *C*. *gloeosporioides* isolates; but CG22 marker detected high heterozygosity of 0.865. Nine SSR loci had no heterozygosity while six displayed less than 0.01 heterozygosity. The expected heterozygosity ranged from 0.042 to 0.554 (Table 3). The Hardy-Weinberg exact test for all populations revealed that 21 loci (95.65%) exhibited significant deviation from HWE corrected for multiple comparisons (*P<*0.001), having levels of heterozygosity less than expected. Two loci (CG22 and CG23) did not show significant departure from HWE, and CG22 had a level of heterozygosity higher than expected. The Fisher’s exact test showed that 60% of the pairwise combinations had significant genotypic linkage disequilibrium using Bonferroni correction. Given that most of the test isolates of *C*. *gloeosporioides* used in this study were assumed to be obtained from asexual populations, these results were not surprising.

**Table 3:** Diversity indices of the microsatellite loci used in the study.

SSR loci	MAF	Na	N_G_	N_E_	R_S_	R_P_	GD	H_O_	H_E_	PIC
CG1	0.91	3	3	1.1	1.46	0.04	0.160	0.000	0.055	0.153
CG2	0.89	3	3	1.1	1.25	0.25	0.200	0.000	0.048	0.186
CG3	0.72	5	7	1.5	2.75	0.11	0.452	0.012	0.319	0.427
CG4	0.50	5	9	2.1	2.86	0.26	0.588	0.043	0.507	0.506
CG6	0.92	5	5	1.2	1.73	0.73	0.151	0.000	0.118	0.149
CG7	0.85	6	7	1.3	2.22	0.74	0.277	0.012	0.205	0.268
CG9	0.85	7	7	1.2	2.26	0.98	0.274	0.000	0.135	0.260
CG11	0.64	8	9	1.9	3.03	1.07	0.537	0.037	0.431	0.494
CG14	0.83	4	5	1.3	2.24	0.03	0.299	0.006	0.199	0.285
CG16	0.46	5	6	2.2	2.72	0.28	0.611	0.006	0.541	0.533
CG18	0.89	5	6	1.1	1.93	0.39	0.198	0.006	0.088	0.189
CG20	0.49	4	5	1.7	2.24	0.24	0.630	0.018	0.407	0.561
CG21	0.43	5	6	2.0	2.57	0.26	0.623	0.018	0.495	0.546
CG22	0.44	6	7	2.3	2.89	0.56	0.626	0.865	0.554	0.552
CG23	0.88	5	5	1.0	1.55	0.55	0.219	0.000	0.042	0.202
CG27	0.44	5	5	1.8	2.39	0.39	0.636	0.000	0.436	0.561
CG28	0.75	5	6	1.4	2.34	0.47	0.417	0.006	0.241	0.386
CG29	0.94	4	4	1.1	1.48	0.48	0.106	0.000	0.099	0.104
CG30	0.41	6	7	2.0	2.75	0.75	0.664	0.006	0.471	0.597
CG32	0.49	5	6	2.2	2.82	0.17	0.599	0.025	0.529	0.520
CG33	0.83	7	7	1.2	2.21	0.98	0.296	0.006	0.125	0.277
CG36	0.86	6	6	1.1	1.93	0.93	0.252	0.000	0.103	0.237
CG37	0.49	4	4	1.6	2.25	0.25	0.620	0.000	0.363	0.547
Mean	0.69	5.13	5.87	1.54	2.26	0.48	0.410	0.046	0.283	0.371

MAF = major allele frequency; N_G_ = number of genotypes, Na = number of alleles; N_E_ = number of effective alleles; R_S_ = allelic richness; R_P_ = private allelic richness; GD = gene diversity; H_O_ = observed heterozygosity; H_E_ = expected heterozygosity; and PIC = polymorphism information content

### Population genetic diversity

The genetic diversity indices for the four *C*. *gloeosporioides* populations are summarized in Table 4. The number of different alleles (Na), private alleles (N_P_) and effective alleles (N_E_) averaged across all loci ranged from 1.70 to 3.26, 0.043 to 1.261, and 1.38 to 1.85, respectively for the four populations (Northwest, Central, Southwest and South Ethiopian). The South Ethiopian (SE) population had the highest while the Northwest Ethiopian (NWE) population had the lowest Na, N_P_ and N_E_ values. Similarly, the SE (R_S_ = 3.16) and NWE (R_S_ = 1.62) populations had the highest and the lowest allelic richness over all pairs of loci, respectively. Private allelic richness was also the least in NWE (R_P_ = 0.04) and the highest in SE (R_P_ = 1.24) *C*. *gloeosporioides* populations. Average observed heterozygosity (H_O_) was in the range of 0.046 to 0.058, with a mean of 0.052 across all loci. Gene diversity was the lowest in NWE population (H_E_ = 0.209) and the highest in SE population (H_E_ = 0.403), and its value averaged over all populations and loci was 0.283 (SE = 0.022). The percentage of polymorphic loci (PL) ranged from 60.87% (NWE) to 100% (SE), with an average of 82.61%. Based on the Shannon's Index (I), higher diversity was observed in SE (I = 0.725) population, but it was least in NWE (I = 0.311) population. The Shannon's Index is an information statistic index, which assumes all types are represented in a sample and that they are randomly sampled. It combines both evenness and richness in a single measure [101,102].

**Table 4:** Summary of the population diversity indices averaged over 23 loci.

Population*	Na	N_P_	N_E_	R_S_	R_P_	PL	H_O_	H_E_	I
CE	2.04	0.130	1.42	1.87	0.13	73.91	0.058	0.234	0.368
NWE	1.70	0.043	1.38	1.62	0.04	60.87	0.048	0.209	0.311
SE	3.26	1.261	1.85	3.16	1.24	100	0.055	0.403	0.725
SWE	2.52	0.522	1.52	2.37	0.49	95.65	0.046	0.287	0.491
Overall	2.38	0.489	1.54	2.26	0.48	82.61	0.052	0.283	0.474

* CE = Central Ethiopia, NWE = Northwest Ethiopia, SE = South Ethiopia, and SWE = Southwest Ethiopia

Na = number of different alleles; N_P_ = number of private alleles; N_E_ = number of effective alleles; R_S_ = allelic richness; R_P_ = private allelic richness; PL = percentage of polymorphic loci; H_O_ = average observed heterozygosity, H_E_ = expected heterozygosity or gene diversity, and I = Shannon's Information Index

### Population genetic structure and gene flow

Analysis of molecular variance (AMOVA) showed that 84% of the total variation was due to differences among isolates within populations, 11% was due to heterozygosity within isolates, and the variation among populations accounted only 5% of the total variation (Table 5). The genetic differentiation among populations (F_ST_ = 0.049 at *P*< 0.001) was significant as indicated by the randomization test. Pairwise F_ST_ values of the genetic distance among all populations were significant (*P*< 0.01) (Table 6). The average estimate of gene flow among populations (N_m_) was 4.8. Gene flow is the movement of genes into or out of a population. Such movement may be due to the movement of gametes, or migration of individuals and even entire population. In the absence of natural selection and genetic drift, gene flow leads to genetic homogeneity among populations as it can introduce new alleles into a population. However, restricted gene flow promotes population divergence via selection and drift, which can lead to speciation [103].

**Table 5:** Analysis of molecular variance among and within populations, and within individuals of *Colletotrichum gloeosporioides* populations based on 23 SSR loci.

Source of variation	Degree of freedom	Sum of squares	Mean squares	Estimated variance	Variation (%)	P value
Among Populations	3	83.428	27.809	0.238	5	<0.001
Among Individuals	159	1367.517	8.601	4.033	84	<0.001
Within Individuals	163	87.000	0.534	0.534	11	<0.001
Total	325	1537.945	4.732			

**Table 6:** Pairwise genetic distance based on F_ST_ matrix, a measure of divergence among the *Colletotrichum* species populations.

Population	CE	NWE	SE
NWE	0.062**		
SE	0.022*	0.056**	
SWE	0.056**	0.067**	0.027*

* significant at *P*<0.01

** significant at *P*< 0.001

**Fig 1:**
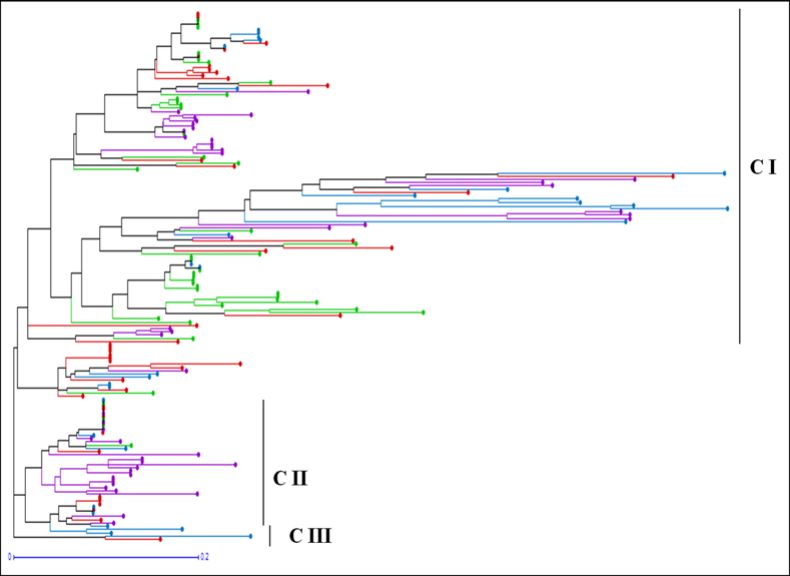
Unweighted Neighbor-joining tree using the simple matching similarity coefficient based on 23 microsatellite markers for the 163 isolates of *Colletotrichum gloeosporioides* isolated from citrus in Ethiopia. The tree shows the clustering pattern of isolates from the four *C*. *gloeosporioides* populations. The populations are color coded as follows: Central region (*red*), Northwest region (*green*), South region (*blue*) and Southwest region (*violet*).

The unweighted Neighbor-joining dendrogram grouped the 163 isolates of the four populations into three major clusters (Fig 1). Of the 163 isolates, 119, 42 and 2 isolates were grouped together in Cluster I, II and III, respectively. Overall topology of the dendrogram indicated the presence of three lineages in *C*. *gloeosporioides* complex associated with citrus leaf and fruit disease in Ethiopia. Several subgroups were observed for populations indicating genetic variability within and among isolates in each population. In terms of locations-specific alleles among the isolates, 17 SSR loci (CG2, CG4, CG6, CG7, CG9, CG11, CG16, CG18, CG20, CG22, Cg23, CG27, CG29, CG30, CG33, CG36 and CG37) showed unique alleles for the isolates from SE, 12 SSR marker (CG6, CG7, CG9, CG11, CG21, CG22, CG23, CG28, CG29, CG30, CG33 and CG36) displayed unique alleles for the isolates from SWE, three SSR markers (CG23, CG27 and CG32) detected unique alleles for the isolates from CE, and only one SSR marker (CG11) detected a unique allele for the isolates from NWE.

**Fig 2:**
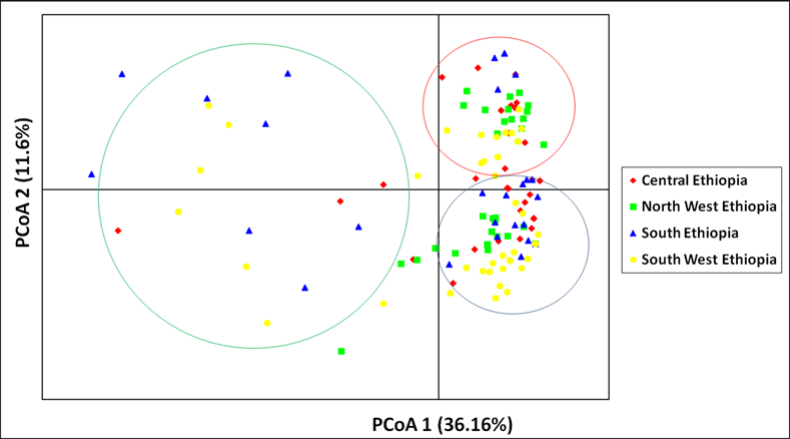
Principal coordinates analysis (PCoA) bi-plot showing the clustering of the 163*Colletotrichumgloeosporioides* isolates based on 23 microsatellite loci. The four populations are color coded as follows: Central region (*red*), Northwest region (*green*), South region (*blue*) and Southwest region (*yellow*). Percentages of variation explained by the first 3 axes (1, 2, and 3) are 36.16%, 11.6% and 8.68%, respectively.

**Fig 3:**
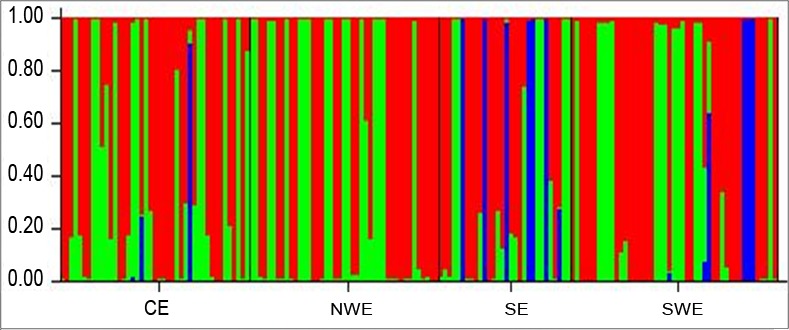
Bayesian model-based estimation of population structure (*K* = 3) for the 163 *Colletotrichum gloeosporioides* isolates in four pre-determined populations (x-axis): Central Ethiopia (CE), Northwest Ethiopia (NEW), South Ethiopia (SE) and Southwest Ethiopia (SWE). Each group is separated by a black vertical line. Numbers in the y-axis show coefficient of membership/assignment.

The pattern of clustering was similar to the Principal coordinate analysis (PCoA) based on the 23 microsatellite loci (Fig 2). Evanno *et al*. [94] method on STRUCTURE outputs predicted *K* = 3 to be the most likely number of clusters (Fig 3).

## Discussion

A large number of hosts are affected by *Colletotrichum* diseases worldwide, but SSR markers are available for a few *Colletotrichum* species such as *C*. *capsici*, associated with chili pepper anthracnose disease [104], and *C*. *acutatum*, the causal agent of post-bloom fruit drop on citrus [105]. The present study is the first report on *C*. *gloeosporioides* SSR markers development and their uses for genetic diversity and population structure study. Information on pathogen genetic diversity and population structure on temporal and spatial scales are important to understand the potential of pathogen populations to spread and overcome host resistance [104,106]. Areas of high biodiversity may serve as a source for the emergence of new genotypes with novel biological characteristics, including changes in pathogen fitness or resistance to certain fungicides [20].

In this study, 23 polymorphic SSR markers were developed and evaluated for assaying the genetic diversity of *C*. *gloeosporioides* isolates from citrus. The polymorphisms detected by these SSR markers were in the range of slightly informative to highly informative. They displayed allelic diversity among the isolates, with 3 to 8 alleles per locus, and the PIC values ranged from 0.104 to 0.597. The PIC provides an estimate of the discriminatory power of a locus by taking into account the number and the relative frequencies of the alleles [24]. All the 23 loci displayed differences for observed heterozygosity, with loci CG22 showing the highest H_O_ at 0.865 and several loci with the lowest value at zero. Higher number of private alleles was observed in the isolates from the South region. Private alleles, or alleles that are unique to certain species or geographic area, are useful in comparing diversity between species or population [107]. The AMOVA results indicated that the highest percentage of variation (84%) was within populations of *C*. *gloeosporioides* isolates. However, the gene diversity observed among the Ethiopian *C*. *gloeosporioides* populations was low. This might be attributed to the recent introduction of the fungus probably of a few genotypes. Detection of high regional genetic diversity within the south Ethiopian population can be explained by the earlier introduction of the disease (in late 1980s) in the region [108]. Similarly, the diversity was also higher within the southwest population where the disease was reported in 1990 [4]. The low genetic diversity in the populations from central and northwest regions can be explained by recent introduction of the disease in these regions [3,109]. These differences may also be due to environmental conditions, geography, and differences in alternative host species diversity that may have a role in generating variability within populations [110]. Ranathunge *et al*. [104] analyzed the genetic diversity of *C*. *capsici* isolates using 27 sequence-tagged microsatellite site markers and found the highest gene diversity of 0.857 at a locus with up to 18 alleles among all the isolates and the differentiation ranged from 0.05 to 0.45. Ciampi *et al*. [105] reported that SSR markers showed better discriminatory power in comparison to the commonly used markers such as the internal transcribed spacers (ITS), the intron 2 of the glyceraldehyde-3-phosphate dehydrogenase gene (G3PD), and the glutamine synthase intron 2 (GS) to estimate genetic diversity in *C*. *acutatum*. The authors found from 3 to 6 alleles per locus, and heterozygosity ranging from 0.093 to 0.590 across loci using nine polymorphic SSR markers.

Various factors can affect genetic diversity [111]. Mutation, population gene flow, and sexual and asexual recombination are the main mechanisms by which genetic diversity can be generated in populations of pathogenic microorganisms [24]. Pathogens showing sexual reproduction pose a greater risk than when the reproductive pattern leads to inbreeding, because new genotypes emerge during the sexual cycle [106]. However, gene diversity may not be influenced by the mode of reproduction because clonally reproducing fungi may show as many alleles as those that undergo recombination [112]. Diversity in fungal populations can arise from transposable elements and mitotic reciprocal translocation events [113]. Genetic diversity can result from accumulation of mutations that can create variations within a species or population [114]. Rampersad [20] suggested that high genetic diversity may be a function of population size. Larger and older populations have and maintain higher levels of gene diversity compared to a recently colonized habitat. In older population, sufficient time has passed to allow mutational events to introduce new genetic variants and for genetic drift to increase the frequencies of these alleles to quantitative levels [20]. McDonald [112] indicated that isolates located at or near the center of origin of the species would have higher level of gene diversity than isolates at other locations because the original population is older. Unique pathogen genotypes may also occupy particular geographical areas that may be associated with host coevolution and adaptation [115]. Weeds *et al*. [116] demonstrated that genetic diversity of *C*. *gloeosporioides* isolates is high where native or naturalized host species occur compared with locations where the host species has been recently introduced.

An insight into the structure of *C*. *gloeosporioides* populations from different locations is valuable in enhancing the understanding of the biology of the pathogen and potentially adaptive genotypic diversity in the species. The *C*. *gloeosporioides* isolates from the four geographic regions of Ethiopia are closely related to one another as reflected by the high genetic identity among populations. Population genetic analyses supported subdivision of the Ethiopian *C*. *gloeosporioides* isolates into three inferred sub-populations. STRUCTURE analysis, PCoA, and the unweighted Neighbor-joining algorithm indicated admixture among the three populations. Geographical separation of isolates into distinctly isolated subpopulations was not observed as evidenced by admixture among isolates from the different regions. The relatively low F_ST_ value (0.049) between the *C*. *gloeosporioides* populations evaluated in this study indicated low differentiation among the groups that might be attributed to gene flow among regions, which is reflected by the high migration rate (N_m_ = 4.8) estimates. A low degree of differentiation in populations of *C*. *gloeosporioides* shown in this study may be attributed to the dispersal of the clonal inoculum over long distances that may allow for pathogen spread in citrus-growing areas in Ethiopia. The citrus fruit produced in the south and southwest parts of Ethiopia are transported to the central areas for marketing while those produced in the northwest are consumed in the same region. The wind direction in Ethiopia is generally from northeast to southwest during the dry season and *vice versa* during the rainy season. These may cause migration and gene flow between populations that resulted in admixture among isolates from the different geographic origin. The pathogen is cosmopolitan in distribution, and conidia are known to be disseminated over long distances by wind [117] and via the movement of infected fruits [118]. Abang *et al*. [119] reported that *C*. *gloeosporioides* infecting yam in Nigeria inferred by 51 microsatellite loci was described as a single population with low intra-population differentiation, high genetic diversity, and evidence of high gene flow. Conversely, there was significant and high genetic differentiation and gene diversity of subpopulations of *C*. *gloeosporioides* infecting strawberry in the United States based on 40 RAPD or microsatellite markers [120].

Pathogen population divergence may occur as a result of genetic drift and local adaptation to increase relative fitness in local environments [121]. McDermott and McDonald [122] indicated that if N_m_ is greater than one, there will be little differentiation among populations and migration is more important than genetic drift. Similarly, Wright [86] stated that the movement of as few as one individual per generation is sufficient to prevent significant divergence between populations. Mechanisms that enable gene flow may act randomly and may be a result of a combination of anthropogenic activities, such as movement and exchange of infected plant material, the process of extinction and recolonization (balance between genetic drift and migration), and alternate hosts outside of the growing season that may allow certain genotypes to persist and undergo expansion in the field [25,106,112,114,122]. For asexually reproducing fungi such as *C*. *gloeosporioides*, identification of population subdivision within a particular geographic area can be associated with the epidemiology of the disease, such as sources of inoculum and host or tissue specificity [25,120].

In conclusion, the microsatellite markers developed in this study were useful to comprehend the genetic diversity and population structure of *C*. *gloeosporioides* isolates from citrus growing regions of Ethiopia. Despite regional differences, the observed genetic diversity in all four populations was lower than expected suggesting inter-regional exchanges of planting materials and dispersal of inoculum among the regions. Information generated in this study could be useful in understanding the pathogen biology and provide basis for other studies on disease development, host-pathogen interaction, and developing disease management strategies including development and use of resistant citrus varieties for citrus leaf and fruit spot. The SSR markers could be useful to characterize *C*. *gloeosporioides* isolates that infect other fruit crops.

## Supporting Information

S1 Table The *Colletotrichum gloeosporioides* isolates used in this study and their geographic origin in Ethiopia. (DOCX)

S2 Table List of microsatellite primers designed for *Colletotrichum gloeosporioides*. (DOCX)

S3 Table Multiplex polymerase chain reactions of *Colletotrichum gloeosporioides* SSR loci. (DOCX)
