# Supplementary material for: Development of Microsatellite Markers and Analysis of Genetic Diversity and Population Structure of Colletotrichum gloeosporioides from Ethiopia
Source: PLoS One. 2016 Mar 15;11(3):e0151257. doi: 10.1371/journal.pone.0151257 (PMC4792483; doi:10.1371/journal.pone.0151257)
Supplement: S1 Table — (DOCX) [file pone.0151257.s001.docx]

S1 Table. The *Colletotrichum gloeosporioides* isolates used in this study and their geographic origin in Ethiopia

| Isolate  Code | Geographic  Region | District | Host | Plant  Part | Date of  Collection | Latitude-Longitude |
| --- | --- | --- | --- | --- | --- | --- |
| ETHCTR001 | Southwest Ethiopia | Ginbo | *Citrus. sinensis* | Leaf | 20-Mar-13 | 7.333 N 36.167 E |
| ETHCTR002 | Central Ethiopia | Kebena | *C. sinensis* | Fruit | 22-Mar-13 | 8.279 N 37.790 E |
| ETHCTR003 | Central Ethiopia | Geta | *C. sinensis* | Fruit | 26-May-14 | 7.876 N 38.034 E |
| ETHCTR004 | Central Ethiopia | Abeshege | *C. sinensis* | Fruit | 27-May-14 | 8.268 N 37.741 E |
| ETHCTR006 | South Ethiopia | Damot Pulasa | *C. sinensis* | Leaf | 25-May-14 | 7.035 N 37.914 E |
| ETHCTR007 | Central Ethiopia | Abeshege | *C. sinensis* | Fruit | 27-May-14 | 8.285 N 37.661 E |
| ETHCTR008 | Southwest Ethiopia | Debre Werk | *C. sinensis* | Leaf | 19-Mar-13 | 6.904 N 35.593 E |
| ETHCTR009 | Southwest Ethiopia | Mana | *C. sinensis* | Leaf | 19-Mar-13 | 7.756 N 36.776 E |
| ETHCTR012 | Southwest Ethiopia | Shebe Senbo | *C. sinensis* | Leaf | 20-Mar-13 | 7.506 N 36.514 E |
| ETHCTR013 | Central Ethiopia | Abeshege | *C. sinensis* | Leaf | 22-Mar-13 | 8.268 N 37.741 E |
| ETHCTR014 | Central Ethiopia | Abeshege | *C. sinensis* | Leaf | 27-May-14 | 8.268 N 37.741 E |
| ETHCTR016 | Central Ethiopia | Wolisso | *C. aurantium* | Leaf | 28-May-14 | 8.533 N 37.967 E |
| ETHCTR017 | Central Ethiopia | Wolisso | *C. aurantium* | Fruit | 28-May-14 | 8.533 N 37.967 E |
| ETHCTR018 | Central Ethiopia | Wolisso | *C. sinensis* | Leaf | 28-May-14 | 8.533 N 37.967 E |
| ETHCTR019 | Central Ethiopia | Wolisso | *C. aurantium* | Leaf | 28-May-14 | 8.533 N 37.967 E |
| ETHCTR020 | Central Ethiopia | Abeshege | *C. sinensis* | Leaf | 27-May-14 | 8.285 N 37.661 E |
| ETHCTR021 | South Ethiopia | Damot Pulasa | *C. sinensis* | Leaf | 25-May-14 | 7.035 N 37.914 E |
| ETHCTR022 | South Ethiopia | Damot Pulasa | *C. sinensis* | Leaf | 25-May-14 | 7.035 N 37.914 E |
| ETHCTR023 | South Ethiopia | Damot Pulasa | *C. sinensis* | Leaf | 25-May-14 | 7.043 N 37.908 E |
| ETHCTR024 | Central Ethiopia | Cheha | *C. sinensis* | Leaf | 26-May-14 | 8.170 N 37.868 E |
| ETHCTR025 | Central Ethiopia | Cheha | *C. sinensis* | Leaf | 26-May-14 | 8.170 N 37.868 E |
| ETHCTR026 | Central Ethiopia | Cheha | *C. sinensis* | Leaf | 26-May-14 | 8.182 N 37.809 E |
| ETHCTR027 | Central Ethiopia | Cheha | *C. sinensis* | Leaf | 26-May-14 | 8.182 N 37.809 E |
| ETHCTR028 | Central Ethiopia | Cheha | *C. sinensis* | Leaf | 26-May-14 | 8.182 N 37.809 E |
| ETHCTR029 | Central Ethiopia | Cheha | *C. sinensis* | Leaf | 26-May-14 | 8.182 N 37.809 E |
| ETHCTR031 | South Ethiopia | Damot Pulasa | *C. sinensis* | Leaf | 25-May-14 | 7.043 N 37.908 E |
| ETHCTR032 | South Ethiopia | Boloso Sore | *C. sinensis* | Leaf | 25-May-14 | 7.096 N 37.709 E |
| ETHCTR033 | South Ethiopia | Boloso Sore | *C. sinensis* | Fruit | 25-May-14 | 7.096 N 37.709 E |
| ETHCTR034 | South Ethiopia | Boloso Sore | *C. sinensis* | Fruit | 25-May-14 | 7.096 N 37.709 E |
| ETHCTR037 | Central Ethiopia | Sekoru | *C. sinensis* | Fruit | 19-Jun-12 | 8.248 N 37.540 E |
| ETHCTR038 | Central Ethiopia | Sekoru | *C. reticulata* | Leaf | 19-Jun-12 | 8.248 N 37.540 E |
| ETHCTR039 | South Ethiopia | Abaya | *C. sinensis* | Leaf | 13-Feb-13 | 6.417 N 38.308 E |
| ETHCTR041 | Northwest Ethiopia | Jabitehnan | *C. reticulata* | Leaf | 27-May-13 | 10.700 N 37.267 E |
| ETHCTR042 | Northwest Ethiopia | Jabitehnan | *C. sinensis* | Leaf | 27-May-13 | 10.700 N 37.267 E |
| ETHCTR043 | Northwest Ethiopia | Jabitehnan | *C. sinensis* | Fruit | 27-May-13 | 10.700 N 37.267 E |
| ETHCTR044 | Northwest Ethiopia | Jabitehnan | *C. sinensis* | Fruit | 27-May-13 | 10.700 N 37.267 E |
| ETHCTR045 | Southwest Ethiopia | Mana | *C. sinensis* | Leaf | 21-Mar-13 | 7.756 N 36.776 E |
| ETHCTR046 | Southwest Ethiopia | Mana | *C. sinensis* | Fruit | 21-Mar-13 | 7.756 N 36.776 E |
| ETHCTR047 | Central Ethiopia | Gorro | *C. sinensis* | Leaf | 22-Mar-13 | 8.403 N 37.870 E |
| ETHCTR048 | South Ethiopia | Abaya | *C. aurantium* | Leaf | 13-Feb-13 | 6.417 N 38.308 E |
| ETHCTR049 | Southwest Ethiopia | Gomma | *C. sinensis* | Leaf | 21-Mar-13 | 7.850 N 36.583 E |
| ETHCTR050 | South Ethiopia | Abaya | *C. aurantium* | Leaf | 13-Feb-13 | 6.433 N 37.883 E |
| ETHCTR051 | South Ethiopia | Abaya | *C. sinensis* | Fruit | 13-Feb-13 | 6.417 N 38.308 E |
| ETHCTR052 | Southwest Ethiopia | Gomma | *C. sinensis* | Leaf | 21-Mar-13 | 7.850 N 36.583 E |
| ETHCTR053 | Central Ethiopia | Abeshege | *C. sinensis* | Leaf | 22-Mar-13 | 8.266 N 37.725 E |
| ETHCTR054 | Southwest Ethiopia | Gomma | *C. sinensis* | Fruit | 21-Mar-13 | 7.850 N 36.583 E |
| ETHCTR055 | Southwest Ethiopia | Mana | *C. sinensis* | Leaf | 21-Mar-13 | 7.756 N 36.776 E |
| ETHCTR056 | South Ethiopia | Abaya | *C. sinensis* | Leaf | 13-Feb-13 | 6.417 N 38.308 E |
| ETHCTR057 | South Ethiopia | Abaya | *C. sinensis* | Leaf | 13-Feb-13 | 6.417 N 38.308 E |
| ETHCTR058 | Central Ethiopia | Abeshege | *C. sinensis* | Fruit | 22-Mar-13 | 8.266 N 37.725 E |
| ETHCTR059 | Northwest Ethiopia | Guangua | *C. sinensis* | Leaf | 26-May-13 | 10.950 N 36.500 E |
| ETHCTR060 | Northwest Ethiopia | Guangua | *C. sinensis* | Leaf | 26-May-13 | 10.950 N 36.500 E |
| ETHCTR061 | Northwest Ethiopia | Jabitehnan | *C. sinensis* | Fruit | 27-May-13 | 10.700 N 37.267 E |
| ETHCTR062 | Northwest Ethiopia | Jabitehnan | *C. sinensis* | Fruit | 27-May-13 | 10.700 N 37.267 E |
| ETHCTR063 | South Ethiopia | Abaya | *C. sinensis* | Leaf | 13-Feb-13 | 6.417 N 38.308 E |
| ETHCTR064 | South Ethiopia | Abaya | *C. sinensis* | Fruit | 13-Feb-13 | 6.417 N 38.308 E |
| ETHCTR065 | Central Ethiopia | Kebena | *C. sinensis* | Fruit | 22-Mar-13 | 8.279 N 37.790 E |
| ETHCTR066 | Central Ethiopia | Kebena | *C. sinensis* | Fruit | 22-Mar-13 | 8.279 N 37.790 E |
| ETHCTR067 | Southwest Ethiopia | Debre Werk | *C. sinensis* | Fruit | 19-Mar-13 | 6.904 N 35.593 E |
| ETHCTR068 | Southwest Ethiopia | Debre Werk | *C. sinensis* | Leaf | 19-Mar-13 | 6.904 N 35.593 E |
| ETHCTR069 | Southwest Ethiopia | Debre Werk | *C. sinensis* | Leaf | 19-Mar-13 | 6.904 N 35.593 E |
| ETHCTR070 | Central Ethiopia | Kebena | *C. sinensis* | Leaf | 22-Mar-13 | 8.279 N 37.790 E |
| ETHCTR071 | Central Ethiopia | Kebena | *C. sinensis* | Leaf | 22-Mar-13 | 8.279 N 37.790 E |
| ETHCTR072 | Northwest Ethiopia | Jabitehnan | *C. reticulata* | Fruit | 27-May-13 | 10.700 N 37.267 E |
| ETHCTR073 | Northwest Ethiopia | Jabitehnan | *C. reticulata* | Fruit | 27-May-13 | 10.700 N 37.267 E |
| ETHCTR074 | South Ethiopia | Aleta Wendo | *C. sinensis* | Leaf | 14-Feb-13 | 6.600 N 38.417 E |
| ETHCTR075 | South Ethiopia | Aleta Wendo | *C. sinensis* | Leaf | 14-Feb-13 | 6.600 N 38.417 E |
| ETHCTR077 | Northwest Ethiopia | Guangua | *C. sinensis* | Leaf | 26-May-13 | 10.950 N 36.500 E |
| ETHCTR078 | Central Ethiopia | Abeshege | *C. sinensis* | Fruit | 27-May-14 | 8.280 N 37.692 E |
| ETHCTR081 | Central Ethiopia | Abeshege | *C. sinensis* | Fruit | 22-Mar-13 | 8.266 N 37.725 E |
| ETHCTR082 | Central Ethiopia | Abeshege | *C. sinensis* | Fruit | 22-Mar-13 | 8.266 N 37.725 E |
| ETHCTR084 | Southwest Ethiopia | Ginbo | *C. sinensis* | Leaf | 20-Mar-13 | 7.333 N 36.167 E |
| ETHCTR085 | Southwest Ethiopia | Ginbo | *C. sinensis* | Leaf | 20-Mar-13 | 7.333 N 36.167 E |
| ETHCTR086 | Southwest Ethiopia | Shebe Senbo | *C. sinensis* | Leaf | 20-Mar-13 | 7.506 N 36.514 E |
| ETHCTR088 | Northwest Ethiopia | Jabitehnan | *C. sinensis* | Leaf | 27-May-13 | 10.700 N 37.267 E |
| ETHCTR089 | Southwest Ethiopia | Mana | *C. sinensis* | Fruit | 21-Mar-13 | 7.756 N 36.776 E |
| ETHCTR090 | Northwest Ethiopia | Jabitehnan | *C. sinensis* | Leaf | 27-May-13 | 10.700 N 37.267 E |
| ETHCTR091 | Northwest Ethiopia | Jabitehnan | *C. sinensis* | Leaf | 27-May-13 | 10.700 N 37.267 E |
| ETHCTR092 | Northwest Ethiopia | Guangua | *C. sinensis* | Fruit | 26-May-13 | 10.950 N 36.500 E |
| ETHCTR093 | Northwest Ethiopia | Guangua | *C. sinensis* | Fruit | 26-May-13 | 10.950 N 36.500 E |
| ETHCTR094 | South Ethiopia | Abaya | *C. sinensis* | Leaf | 13-Feb-13 | 6.417 N 38.308 E |
| ETHCTR095 | Northwest Ethiopia | Guangua | *C. sinensis* | Leaf | 26-May-13 | 10.950 N 36.500 E |
| ETHCTR096 | Southwest Ethiopia | Shebe Senbo | *C. sinensis* | Leaf | 20-Mar-13 | 7.506 N 36.514 E |
| ETHCTR097 | South Ethiopia | Abaya | *C. sinensis* | Fruit | 13-Feb-13 | 6.417 N 38.308 E |
| ETHCTR098 | South Ethiopia | Boloso Sore | *C. sinensis* | Leaf | 26-May-14 | 7.096 N 37.709 E |
| ETHCTR101 | Central Ethiopia | Abeshege | *C. sinensis* | Leaf | 20-Jun-12 | 8.283 N 37.783 E |
| ETHCTR103 | South Ethiopia | Abaya | *C. aurantium* | Leaf | 13-Feb-13 | 6.433 N 37.883 E |
| ETHCTR104 | Southwest Ethiopia | Gomma | *C. sinensis* | Leaf | 21-Mar-13 | 7.850 N 36.583 E |
| ETHCTR105 | Southwest Ethiopia | Gomma | *C. sinensis* | Leaf | 21-Mar-13 | 7.850 N 36.583 E |
| ETHCTR106 | Southwest Ethiopia | Gomma | *C. sinensis* | Leaf | 21-Mar-13 | 7.850 N 36.583 E |
| ETHCTR107 | Southwest Ethiopia | Gomma | *C. sinensis* | Fruit | 21-Mar-13 | 7.850 N 36.583 E |
| ETHCTR108 | Northwest Ethiopia | Jabitehnan | *C. sinensis* | Leaf | 27-May-13 | 10.700 N 37.267 E |
| ETHCTR109 | Northwest Ethiopia | Jabitehnan | *C. sinensis* | Leaf | 27-May-13 | 10.700 N 37.267 E |
| ETHCTR110 | Northwest Ethiopia | Jabitehnan | *C. sinensis* | Leaf | 27-May-13 | 10.700 N 37.267 E |
| ETHCTR111 | Northwest Ethiopia | Jabitehnan | *C. sinensis* | Fruit | 27-May-13 | 10.700 N 37.267 E |
| ETHCTR113 | Central Ethiopia | Abeshege | *C. sinensis* | Leaf | 20-Jun-12 | 8.283 N 37.783 E |
| ETHCTR115 | South Ethiopia | Aleta Wendo | *C. sinensis* | Leaf | 14-Feb-13 | 6.600 N 38.417 E |
| ETHCTR116 | South Ethiopia | Aleta Wendo | *C. sinensis* | Leaf | 14-Feb-13 | 6.600 N 38.417 E |
| ETHCTR117 | Southwest Ethiopia | Mana | *C. sinensis* | Leaf | 21-Mar-13 | 7.756 N 36.776 E |
| ETHCTR118 | Northwest Ethiopia | Jabitehnan | *C. reticulata* | Fruit | 27-May-13 | 10.700 N 37.267 E |
| ETHCTR119 | Northwest Ethiopia | Jabitehnan | *C. reticulata* | Fruit | 27-May-13 | 10.700 N 37.267 E |
| ETHCTR120 | Northwest Ethiopia | Jabitehnan | *C. reticulata* | Fruit | 27-May-13 | 10.700 N 37.267 E |
| ETHCTR121 | Northwest Ethiopia | Jabitehnan | *C. reticulata* | Fruit | 27-May-13 | 10.700 N 37.267 E |
| ETHCTR122 | Southwest Ethiopia | Ginbo | *C. sinensis* | Leaf | 20-Mar-13 | 7.333 N 36.167 E |
| ETHCTR123 | Southwest Ethiopia | Ginbo | *C. sinensis* | Leaf | 20-Mar-13 | 7.333 N 36.167 E |
| ETHCTR124 | Southwest Ethiopia | Ginbo | *C. sinensis* | Leaf | 20-Mar-13 | 7.333 N 36.167 E |
| ETHCTR127 | Southwest Ethiopia | Shebe Senbo | *C. sinensis* | Leaf | 20-Mar-13 | 7.506 N 36.514 E |
| ETHCTR128 | Southwest Ethiopia | Shebe Senbo | *C. sinensis* | Leaf | 20-Mar-13 | 7.506 N 36.514 E |
| ETHCTR129 | Southwest Ethiopia | Debre Werk | *C. sinensis* | Leaf | 19-Mar-13 | 6.904 N 35.593 E |
| ETHCTR130 | Southwest Ethiopia | Debre Werk | *C. sinensis* | Leaf | 19-Mar-13 | 6.904 N 35.593 E |
| ETHCTR131 | Southwest Ethiopia | Debre Werk | *C. sinensis* | Leaf | 19-Mar-13 | 6.904 N 35.593 E |
| ETHCTR132 | Central Ethiopia | Abeshege | *C. sinensis* | Fruit | 22-Mar-13 | 8.280 N 37.692 E |
| ETHCTR133 | Central Ethiopia | Abeshege | *C. sinensis* | Fruit | 22-Mar-13 | 8.280 N 37.692 E |
| ETHCTR134 | Central Ethiopia | Abeshege | *C. sinensis* | Leaf | 22-Mar-13 | 8.266 N 37.725 E |
| ETHCTR136 | Central Ethiopia | Abeshege | *C. sinensis* | Leaf | 22-Mar-13 | 8.266 N 37.725 E |
| ETHCTR137 | Central Ethiopia | Abeshege | *C. sinensis* | Fruit | 22-Mar-13 | 8.266 N 37.725 E |
| ETHCTR138 | Central Ethiopia | Abeshege | *C. sinensis* | Fruit | 22-Mar-13 | 8.266 N 37.725 E |
| ETHCTR140 | Southwest Ethiopia | Debre Werk | *C. sinensis* | Leaf | 19-Mar-13 | 6.904 N 35.593 E |
| ETHCTR141 | Southwest Ethiopia | Debre Werk | *C. sinensis* | Leaf | 19-Mar-13 | 6.904 N 35.593 E |
| ETHCTR142 | Southwest Ethiopia | Debre Werk | *C. sinensis* | Leaf | 19-Mar-13 | 6.904 N 35.593 E |
| ETHCTR143 | Southwest Ethiopia | Debre Werk | *C. sinensis* | Leaf | 19-Mar-13 | 6.904 N 35.593 E |
| ETHCTR148 | Central Ethiopia | Abeshege | *C. sinensis* | Leaf | 22-Mar-13 | 8.266 N 37.725 E |
| ETHCTR151 | Southwest Ethiopia | Debre Werk | *C. sinensis* | Fruit | 19-Mar-13 | 6.904 N 35.593 E |
| ETHCTR152 | Southwest Ethiopia | Debre Werk | *C. sinensis* | Fruit | 19-Mar-13 | 6.904 N 35.593 E |
| ETHCTR153 | Southwest Ethiopia | Debre Werk | *C. sinensis* | Fruit | 19-Mar-13 | 6.904 N 35.593 E |
| ETHCTR154 | Northwest Ethiopia | Jabitehnan | *C. sinensis* | Fruit | 27-May-13 | 10.700 N 37.267 E |
| ETHCTR156 | South Ethiopia | Abaya | *C. sinensis* | Fruit | 13-Feb-13 | 6.417 N 38.308 E |
| ETHCTR157 | Southwest Ethiopia | Debre Werk | *C. sinensis* | Leaf | 19-Mar-13 | 6.904 N 35.593 E |
| ETHCTR158 | Southwest Ethiopia | Debre Werk | *C. sinensis* | Leaf | 19-Mar-13 | 6.904 N 35.593 E |
| ETHCTR159 | Southwest Ethiopia | Debre Werk | *C. sinensis* | Leaf | 19-Mar-13 | 6.904 N 35.593 E |
| ETHCTR160 | Southwest Ethiopia | Gomma | *C. sinensis* | Leaf | 21-Mar-13 | 7.850 N 36.583 E |
| ETHCTR161 | Northwest Ethiopia | Guangua | *C. sinensis* | Leaf | 26-May-13 | 10.950 N 36.500 E |
| ETHCTR162 | Northwest Ethiopia | Jabitehnan | *C. sinensis* | Fruit | 27-May-13 | 10.700 N 37.267 E |
| ETHCTR163 | Northwest Ethiopia | Jabitehnan | *C. sinensis* | Leaf | 27-May-13 | 10.700 N 37.267 E |
| ETHCTR164 | Northwest Ethiopia | Jabitehnan | *C. sinensis* | Leaf | 27-May-13 | 10.700 N 37.267 E |
| ETHCTR165 | Northwest Ethiopia | Jabitehnan | *C. sinensis* | Leaf | 27-May-13 | 10.700 N 37.267 E |
| ETHCTR166 | South Ethiopia | Boloso Sore | *C. sinensis* | Fruit | 26-May-14 | 7.096 N 37.709 E |
| ETHCTR167 | South Ethiopia | Boloso Sore | *C. sinensis* | Fruit | 26-May-14 | 7.096 N 37.709 E |
| ETHCTR169 | South Ethiopia | Boloso Sore | *C. sinensis* | Fruit | 26-May-14 | 7.096 N 37.709 E |
| ETHCTR170 | Southwest Ethiopia | Ginbo | *C. sinensis* | Leaf | 20-Mar-13 | 7.333 N 36.167 E |
| ETHCTR172 | Southwest Ethiopia | Ginbo | *C. sinensis* | Leaf | 20-Mar-13 | 7.333 N 36.167 E |
| ETHCTR173 | Southwest Ethiopia | Mana | *C. sinensis* | Leaf | 21-Mar-13 | 7.756 N 36.776 E |
| ETHCTR174 | Southwest Ethiopia | Mana | *C. sinensis* | Leaf | 21-Mar-13 | 7.756 N 36.776 E |
| ETHCTR175 | Northwest Ethiopia | Jabitehnan | *C. reticulata* | Leaf | 27-May-13 | 10.700 N 37.267 E |
| ETHCTR178 | Northwest Ethiopia | Jabitehnan | *C. reticulata* | Leaf | 27-May-13 | 10.700 N 37.267 E |
| ETHCTR179 | Northwest Ethiopia | Jabitehnan | *C. reticulata* | Leaf | 27-May-13 | 10.700 N 37.267 E |
| ETHCTR180 | Northwest Ethiopia | Jabitehnan | *C. reticulata* | Leaf | 27-May-13 | 10.700 N 37.267 E |
| ETHCTR181 | Northwest Ethiopia | Jabitehnan | *C. sinensis* | Fruit | 27-May-13 | 10.700 N 37.267 E |
| ETHCTR182 | Northwest Ethiopia | Jabitehnan | *C. sinensis* | Fruit | 27-May-13 | 10.700 N 37.267 E |
| ETHCTR183 | Northwest Ethiopia | Jabitehnan | *C. sinensis* | Fruit | 27-May-13 | 10.700 N 37.267 E |
| ETHCTR184 | Northwest Ethiopia | Jabitehnan | *C. sinensis* | Fruit | 27-May-13 | 10.700 N 37.267 E |
| ETHCTR185 | Northwest Ethiopia | Jabitehnan | *C. sinensis* | Fruit | 27-May-13 | 10.700 N 37.267 E |
| ETHCTR186 | Northwest Ethiopia | Jabitehnan | *C. sinensis* | Fruit | 27-May-13 | 10.700 N 37.267 E |
| ETHCTR187 | Central Ethiopia | Cheha | *C. sinensis* | Leaf | 26-May-14 | 8.182 N 37.809 E |
| ETHCTR188 | Central Ethiopia | Geta | *C. sinensis* | Fruit | 26-May-14 | 7.876 N 38.034 E |
| ETHCTR189 | South Ethiopia | Damot Pulasa | *C. sinensis* | Leaf | 26-May-14 | 7.043 N 37.908 E |
| ETHCTR190 | South Ethiopia | Damot Pulasa | *C. sinensis* | Leaf | 26-May-14 | 7.043 N 37.908 E |
| ETHCTR192 | Northwest Ethiopia | Jabitehnan | *C. sinensis* | Leaf | 27-May-13 | 10.700 N 37.267 E |
| ETHCTR193 | Northwest Ethiopia | Jabitehnan | *C. sinensis* | Fruit | 27-May-13 | 10.700 N 37.267 E |
| ETHCTR194 | Central Ethiopia | Abeshege | *C. sinensis* | Leaf | 27-May-14 | 8.285 N 37.661 E |
| ETHCTR197 | Central Ethiopia | Wolisso | *C. aurantium* | Leaf | 28-May-14 | 8.533 N 37.967 E |
| ETHCTR198 | Central Ethiopia | Wolisso | *C. aurantium* | Fruit | 28-May-14 | 8.533 N 37.967 E |
