# Supplementary material for: Development of Microsatellite Markers and Analysis of Genetic Diversity and Population Structure of Colletotrichum gloeosporioides from Ethiopia
Source: PLoS One. 2016 Mar 15;11(3):e0151257. doi: 10.1371/journal.pone.0151257 (PMC4792483; doi:10.1371/journal.pone.0151257)
Supplement: S2 Table — (DOCX) [file pone.0151257.s002.docx]

S2 Table. List of microsatellite primers designed for *Colletotrichum gloeosporioides*

| Locus^a^ | Forward Primer Sequences (5’- 3’) | Reverse Primer Sequences (5’- 3’) | T_a_ (ºC)^b^ | Estimated  Product Size (bp) | Repeat Motifs |
| --- | --- | --- | --- | --- | --- |
| CG1 | 6FAM-CAAGCAGTCTTTCTGGTCTT | AAAACAACTTCTCTCGTCCA | 51 | 129 | (TG)6 |
| CG2 | VIC-TCACCTTCACTCACACTTGA | CTACTTCGAGACAAGCACG | 51 | 200 | (CT)6 |
| CG3 | PET-GGGTTTTCTCATTCTCAACA | CGACATGATCCATAGCAAG | 50 | 249 | (AT)6 |
| CG4 | NED-AACTCAAGATCAAGAGCAGC | ATGTACAGACGCTCACACAA | 51 | 158 | (TG)6 |
| CG5 | 6FAM-GCTCGTACCTACGCAGTAAT | TCATCATGGACAATCATCAC | 54 | 216 | (AC)6 |
| CG6 | VIC-AGAGCAAGACAGGTGGAATA | ATCCCTGACTGCATAAACC | 51 | 221 | (AC)6 |
| CG7 | PET-ATCTCCAGAGAGAACACAGC | GAGACCTCACGGAATTGAC | 51 | 161 | (TG)7 |
| CG8 | NED-CTGCATATCAACCAGCACTA | AAAACAACAAGGACGACAAG | 51 | 156 | (GC)6 |
| CG9 | 6FAM-GTCTTGATGCTGAAGTCCAC | CACTCCTTCATAGAACACCC | 51 | 222 | (TG)6 |
| CG10 | VIC-GAGAAGGCTGACGAAGAAG | GACGGCTTCCGCAACTAC | 54 | 213 | (GC)6 |
| CG11 | PET-CAGTGAAGATAGGGAAGCAG | ACCACTCAGCGTATGAGAAA | 51 | 119 | (GT)8 |
| CG12 | NED-AGACACATCGAAGATGGAAT | TGCCAGAATGTAGTTGTGAA | 51 | 248 | (TG)10 |
| CG13 | 6FAM-GAGGCAATTGAACTCACACT | GAAGTACACCAAGTGCAGGT | 51 | 247 | (CT)6 |
| CG14 | VIC-ACATGACATCAAACCAGCTT | CTCTTGACCCGATGTTCTAT | 51 | 171 | (TC)7 |
| CG15 | PET-GTTTGCATATCCGAGTGC | ACATCCCAGTCACGTTTTAC | 50 | 236 | (TC)7 |
| CG16 | NED-CCATTCTTTGTACTGGTCGT | GACATCAGACATCCATCCTC | 51 | 193 | (TG)6 |
| CG17 | 6FAM-TATACCAGTCCCCTCAACTG | GATCCAGAGTCTCTTATCGC | 51 | 246 | (GA)7 |
| CG18 | VIC-TCCAGACGGATAGCTTACAC | GAGGTATTGCGTCCACTAAG | 51 | 200 | (CT)7 |
| CG19 | PET-AATATCCAAGCCAACTGATG | TGGAGATCTTTACAATCGCT | 50 | 216 | (CA)6 |
| CG20 | NED-CATAGTCCGTCCAGTCTCAT | CTAATGAAAAGTCGTGGAGC | 51 | 234 | (GA)8 |
| CG21 | 6FAM-GTCTCACTCAGTCTCAAGCC | AACACAGTCTGAGAGGCAAT | 51 | 229 | (AT)9 |
| CG22 | VIC-CTTCGAGTCACCTCTTCAAC | CAGAGTGGTAAAGGTGGTGT | 51 | 239 | (AC)7 |
| CG23 | PET-TATTAGATCCCGACCTTGTG | ATCCTGGTCACCATAATCC | 51 | 176 | (GA)6 |
| CG24 | NED-GTATGCGACCTTACGCTTC | TTGACGGGAGACTCTAATTG | 51 | 220 | (TC)6 |
| CG25 | 6FAM-AAGAGCCTCCTCTCGGTAT | AAGTATTTGTCGCCATCAAC | 51 | 235 | (AG)8 |
| CG26 | VIC-CGCATCTTGGATTTCTATTC | TTTCCTCCATCTCAACATTC | 50 | 237 | (GT)8 |
| CG27 | PET-CCTGTTGATCCATGATGTAA | GAAAGGCTGACTTGTGAACT | 50 | 128 | (GT)6 |
| CG28 | NED-CATATCTCTTCGTACCTCGC | GGTTTGTTGTCTGCTTCTCT | 51 | 168 | (AG)8 |
| CG29 | 6FAM-TTTCAACTACATCCCACCTC | GTATTTGAGGCTGAAGCAAG | 51 | 70 | (AC)7 |
| CG30 | VIC-CGTCATTTTCTGGATTCACT | ATCCATTGGGCTGTCCAT | 50 | 158 | (GT)9 |
| CG31 | PET-CAGGATATATTGGACCATGC | CTACTTTTACCGCACACACA | 54 | 158 | (TG)10 |
| CG32 | NED-TTGTTAGCATCGTGAGTCAG | GCAGTTGATTGAGCAGTACA | 51 | 213 | (AG)10 |
| CG33 | 6FAM-GGCATCTATGGACTAGCAGA | TCATACACCAAAGCTTCCTC | 51 | 233 | (GC)6 |
| CG34 | VIC-GGGACTCTCTCTCTTTTCGT | GTGGTGGAAAATCTGTCCTA | 54 | 225 | (TA)6 |
| CG35 | PET-TAAGTCGGGTAATGAATGGT | TGGTGCTCTTCTTACCTACC | 54 | 250 | (GA)6 |
| CG36 | NED-CCACTCAATTCAATGACAGA | TGAGAGAGTTGTGTCCATCA | 50 | 227 | (AC)7 |
| CG37 | 6FAM-TTATATGCCCCATACTCACC | GGGTCATCTTACACCGTTAC | 50 | 234 | (CA)8 |
| CG38 | VIC-TTCTCTTGGAGCTAGACGAC | AGTCATTGACGTGTATGTGC | 50 | 224 | (CT)6 |
| CG39 | PET-ATAAATCAGGTCGTCTGCAT | TAAGAGTGGAAAAGAGCCAA | 54 | 226 | (GT)7 |
| CG40 | NED-GTCTTGACGTTGGGAAAAT | TTGAACAGAGCATTATGACG | 50 | 242 | (CA)8 |
| CG41 | 6FAM-ACGATTGAGTTCTGAAAGGA | AATGTACTCCGTTCCGCC | 50 | 96 | (GA)6 |
| CG42 | VIC-GACTGACGGTGTTGTTCC | GACTTGGAGTGAAGGGAGAT | 54 | 237 | (TC)7 |
| CG43 | PET-AGTTGCTGTAGAACCACCAC | GAGACCGAGACGTTGAGAG | 54 | 131 | (GA)6 |
| CG44 | NED-TCCATCGTCATATTTCCTTC | TTCATGCGTTAGTCAGTTTG | 50 | 188 | (TC)7 |
| CG45 | 6FAM-GGCACCGATAAGATTTTGTA | ACTGGGTCTAACTCGAAACA | 50 | 158 | (CG)6 |
| CG46 | VIC-AGAGACTCAACAGGCATTGT | CTAATCACGAGACCCAACAT | 50 | 242 | (TC)6 |
| CG47 | PET-GGAGTCGAGCACACTACTAGA | CGAATCATCGATAGGCTTAC | 54 | 228 | (GA)6 |
| CG48 | NED-TGATGGAGACGTACACTTGA | GACACCATGCAGAGAAACTT | 54 | 179 | (GA)7 |
| CG49 | 6FAM-AACTGTATCCACCAGAGCC | TAACTCATCATCGACAGCAG | 54 | 149 | (GC)6 |
| CG50 | VIC-AACCACTCCAACAACCAC | CAAGTACTCCTGAAACCCAG | 54 | 136 | (AG)6 |

^a^CG12, CG24, CD38 and CG 41 did not amplify

^b^ T_a_ = Annealing temperature
