# Supplementary material for: Development of Microsatellite Markers and Analysis of Genetic Diversity and Population Structure of Colletotrichum gloeosporioides from Ethiopia
Source: PLoS One. 2016 Mar 15;11(3):e0151257. doi: 10.1371/journal.pone.0151257 (PMC4792483; doi:10.1371/journal.pone.0151257)
Supplement: S3 Table — (DOCX) [file pone.0151257.s003.docx]

S3 Table. Multiplex polymerase chain reactions of *Colletotrichum gloeosporioides* SSR loci

| Multiplex | Locus | Forward Primer per Reaction (µM) | Reverse Primer per Reaction (µM) | Estimated  Product Size (bp) | T_a_ (ºC)^*^ |
| --- | --- | --- | --- | --- | --- |
| 1 | CG1  CG6  CG7  CG16 | 0.05  0.05  0.08  0.08 | 0.05  0.05  0.08  0.08 | 129  221  161  193 | 51 |
| 2 | CG4  CG18  CG21  CG23 | 0.075  0.07  0.05  0.15 | 0.075  0.07  0.05  0.15 | 158  200  229  176 | 51 |
| 3 | CG2  CG8  CG9  CG11 | 0.08  0.08  0.06  0.10 | 0.08  0.08  0.06  0.10 | 200  156  222  119 | 51 |
| 4 | CG20  CG22  CG25  CG28 | 0.06  0.08  0.06  0.08 | 0.06  0.08  0.06  0.08 | 234  239  235  168 | 51 |
| 5 | CG7  CG14  CG29  CG32  CG33 | 0.10  0.08  0.08  0.08  0.08 | 0.10  0.08  0.08  0.08  0.08 | 161  171  70  213  233 | 51 |
| 6 | CG6  CG11  CG13  CG20 | 0.05  0.08  0.07  0.07 | 0.05  0.08  0.07  0.07 | 221  119  247  234 | 51 |
| 7 | CG2  CG17  CG23  CG28 | 0.08  0.15  0.12  0.07 | 0.08  0.15  0.12  0.07 | 200  246  176  168 | 51 |
| 8 | CG3  CG26  CG27  CG36  CG37 | 0.08  0.08  0.06  0.05  0.05 | 0.08  0.08  0.06  0.05  0.05 | 249  237  128  227  234 | 50 |
| 9 | CG15  CG29  CG30  CG44 | 0.12  0.08  0.10  0.10 | 0.12  0.08  0.10  0.10 | 236  70  158  188 | 50 |
| 10 | CG19  CG40  CG45  CG46 | 0.08  0.05  0.05  0.04 | 0.08  0.05  0.05  0.04 | 216  242  158  242 | 50 |
| 11 | CG5  CG10  CG35  CG43  CG48 | 0.07  0.05  0.08  0.15  0.11 | 0.07  0.05  0.08  0.15  0.11 | 216  213  250  131  179 | 54 |
| 12 | CG4  CG34  CG47  CG49 | 0.08  0.05  0.15  0.06 | 0.08  0.05  0.15  0.06 | 158  225  228  149 | 54 |
| 13 | CG31  CG37  CG39  CG42  CG50 | 0.13  0.05  0.12  0.07  0.08 | 0.13  0.05  0.12  0.07  0.08 | 158  234  226  237  136 | 54 |

^*^ T_a_ = Annealing temperature
